# Relationship between hyperhomocysteinemia and coexisting obesity with low skeletal muscle mass in asymptomatic adult population

**DOI:** 10.1038/s41598-022-16401-1

**Published:** 2022-07-20

**Authors:** Tae Kyung Yoo, Hye Chang Rhim, Yong-Taek Lee, Kyung Jae Yoon, Chul-Hyun Park

**Affiliations:** 1 Department of Medicine, MetroWest Medical Center/Tufts University School of Medicine, Framingham, MA USA; 2 Department of Physical and Rehabilitation Medicine, Kangbuk Samsung Hospital, Sungkyunkwan University School of Medicine, 29 Saemunanro, Jongno-gu, Seoul, 03181 Republic of Korea; 3 Medical Research Institute, Kangbuk Samsung Hospital, Sungkyunkwan University School of Medicine, Seoul, Republic of Korea

**Keywords:** Endocrinology, Endocrine system and metabolic diseases, Metabolic disorders

## Abstract

The relationship between hyperhomocysteinemia (HHcy) and obesity with low skeletal muscle mass (LMM) has not been established. We aim to assess the association between HHcy and the coexistence of obesity and LMM in asymptomatic adult population. We conducted a population-based cross-sectional study among asymptomatic individuals who underwent measurements of plasma homocysteine and body composition analysis. HHcy was defined as > 15 umol/L, obesity as body mass index ≥ 25 (kg/m^2^), and LMM as skeletal muscle index less than 2 SD below the sex-specific mean of young adults. The participants were classified into ‘control’, ‘obesity alone’, ‘LMM alone’, and ‘obesity with LMM’. Among 113,805 participants, the prevalence of HHcy was 8.3% in control, 8.7% in obesity alone, 10.0% in LMM alone, and 13.0% in obesity with LMM (*p* for trend < 0.001). In a multivariable logistic regression analysis, the associations showed a positive trend for HHcy along the groups from obesity alone, to LMM alone, and to obesity with LMM. HHcy was independently associated with the presence of LMM alone (adjusted odds ratio 1.186 [95% confidence interval 1.117–1.259]) and obesity with LMM (1.424 [1.134–1.788]), respectively. This study demonstrated that HHcys was more strongly associated with coexistence of obesity and LMM than either condition alone in the adult population.

## Introduction

Sarcopenia is defined as a progressive decline in skeletal muscle mass and function by aging^1^. It has been reported that decline in skeletal muscle mass begins around 45 years of age^2^ and is accelerated in both men and women after 60 years of age, while fat mass continues to increase until around 75 years of age^3^. Low skeletal muscle mass (LMM) has been implicated in several adverse health outcomes including increased risks of falls and fractures^4^, osteoporosis^5^, cardiovascular and pulmonary diseases^6^, and mobility disorders with poor quality of life^7^. When LMM coexists with an increased fat mass, a condition also known as sarcopenic obesity, these two metabolic burdens are thought to potentiate each other and synergize their negative health outcomes^8,9^. Currently, the prevalence of obesity and LMM is increasing in the general population, which makes sarcopenic obesity a new major health issue in worldwide^10^.

Hyperhomocysteinemia is a prevalent condition that poses a significant metabolic burden^11^. Excess Homocysteine can have a detrimental effect on multiple organs by forming active oxygen species and facilitating endothelial, smooth muscle cell dysfunction, leading to coronary heart disease and cerebrovascular disease^12^. Recently, hyperhomocysteinemia (HHcy), which may derive from smoking or alcohol use, poor nutrition, impaired kidney function, or an enzyme deficiency^13^, has gained attention in terms of its association with skeletal muscle, physical performance, and obesity^12^. However, previous studies investigating the association between HHcy and LMM showed conflicting results and focused on older adults^14–18^.

Since sarcopenic obesity has been understood as a multifactorial disease including sedentary behavior that can occur in young and middle-aged populations^19,20^, it is important to understand the association between homocysteine and LMM in these populations. In the light of such importance, our research group assessed the association between HHcy and LMM in adults including both younger and older populations and found that HHcy itself may be an independent risk factor for decline in skeletal muscle mass^12^. Up to date, however, there is limited study on the relationship between HHcy and coexistence of obesity with LMM and whether obesity with LMM is more strongly associated with HHcy than obesity or LMM alone in asymptomatic adults.

The purpose of this study is to investigate the association of HHcy with the presence of obesity alone, LMM alone, and the coexistence of obesity with LMM. We hypothesized that the participants with HHcy would be more strongly associated with obesity and LMM than those without HHcys. In addition, the association between HHcy and the coexistence of obesity with LMM would be stronger than that of HHcy and obesity or LMM alone.

## Results

### Baseline demographic characteristics

The baseline demographic characteristics of the 113,805 eligible participants according to four different groups of body composition are reported in Table 1. Control group was 42.58% (*n* = 48,458), obesity alone group 41.57% (*n* = 47,313), LMM alone group 15.24% (*n* = 17,350), and obesity with LMM group 0.60% (*n* = 684). The mean age of participants was 38.82 ± 10.8 (range: 18 to 92 years). Men were 94.6% (*n* = 107,660). SMI was 7.8 ± 0.6 kg/m^2^ in control, 8.4 ± 0.7 kg/m^2^ in obesity alone, 7.0 ± 0.5 kg/m^2^ in LMM alone, 7.2 ± 0.4 kg/m^2^ in obesity with LMM. Baseline characteristics of each group were significantly different in all variables (*p* < 0.001).

**Table 1:** Baseline demographic characteristics of study participants (*n* = 113,805).

	Total	Control	Obesity alone	LMM alone	Obesity with LMM	*p* value
Number of subjects	113,805	48,458 (42.58%)	47,313 (41.57%)	17,350 (15.24%)	684 (0.60%)	
Age, years	38.32 ± 10.8	37.88 ± 10.5	38.05 ± 10.0	40.1 ± 12.9	44.78 ± 15.42	< 0.001*
Men (n, %)	107,651 (94.6)	44,701 (92.2)	45,620 (96.4)	16,666 (96.1)	664 (97.1)	< 0.001^†^
Height (cm)	172.6 ± 7.1	173.4 ± 7.2	173.1 ± 6.8	169.8 ± 3.4	165.2 ± 6.6	< 0.001*
Weight (kg)	73.9 ± 11.6	69.5 ± 6.9	82.9 ± 9.9	61.4 ± 6.3	70.9 ± 5.7	< 0.001*
BMI (kg/m^2^)	24.7 ± 3.2	23.1 ± 1.3	27.6 ± 2.4	21.3 ± 1.8	25.9 ± 0.9	< 0.001*
Appendicular skeletal muscle mass (kg)	23.8 ± 3.7	23.6 ± 3.2	25.5 ± 3.4	20.2 ± 2.4	19.6 ± 2.1	< 0.001*
SMI (kg/m^2^)	8.0 ± 0.8	7.8 ± 0.6	8.4 ± 0.7	7.0 ± 0.5	7.2 ± 0.4	< 0.001*
Hypertension (n, %)	14,618 (12.8)	4227 (8.7)	8511 (18)	1713 (9.9)	167 (24.4)	< 0.001^†^
Diabetes mellitus (n, %)	4277 (3.8)	1337 (2.8)	2114 (4.5)	767 (4.4)	59 (8.6)	< 0.001^†^
HDL-C (mg/dL)	54.1 ± 13.7	57.1 ± 13.9	49.4 ± 11.8	58.5 ± 14.6	51.2 ± 11.7	< 0.001*
ALT (IU/L)	30.9 ± 25.7	24.7 ± 19.1	39.4 ± 31.2	24.5 ± 16.9	36.7 ± 26.3	< 0.001*
CRP (mg/dL)	0.06 (0.03–0.12)	0.04 (0.02–0.09)	0.08 (0.04–0.17)	0.05 (0.02–0.10)	0.10 (0.05–0.21)	< 0.001*

Data are presented as mean ± standard deviation, number (percentage) or median (interquartile range).

SMI (kg/m^2^) = appendicular skeletal muscle mass (kg)/height (m)^2^.

*ALT* alanine aminotransferase, *BMI* body mass index, *CRP* C-reactive protein, *HDL-C* high-density lipoprotein cholesterol, *LMM* low skeletal muscle mass, *SMI* skeletal muscle mass index.

*p* values for between group difference by *one-way ANOVA in continuous variables or by ^†^Chi-square test in categorical variables.

### Prevalence of hyperhomocysteinemia in control, obesity alone, low muscle mass alone and coexisting obesity with low muscle mass

The prevalence of participants with HHcy was 8.3% in control, 8.7% in obesity alone, 10.0% in LMM alone, and 13.0% in obesity with LMM group, with the obesity with LMM group showing the highest (p < 0.001). Overall, prevalence of HHcy increased from control to obesity alone, to LMM alone, and to obesity with LMM group (*p* for trend < 0.001) (Table 2).

**Table 2:** Prevalence of hyperhomocysteinemia (≥ 15 umol/L) in control, obesity, low skeletal muscle mass, and obesity and low skeletal muscle mass.

	Control	Obesity alone	LMM alone	Obesity with LMM	*p* for trend
**Classification according to homocysteine level**					< 0.001
Normal Hcy (< 15 umol/L) (%)	91.7	91.3	90.0	87.0	
Hyperhomocysteinemia (%)	8.3	8.7	10.0	13.0	

*Hcy* homocysteine, *LMM* low skeletal muscle mass.

### Comparison of natural log transformed homocysteine level between study groups

We conducted an analysis to assess the relationships when Hcy was expressed as a continuous variable. Adjusted means of ln (homocysteine) level in the groups were estimated from ANCOVA after adjustments for age, sex, history of hypertension, history of diabetes, HDL-C, ALT, and CRP. The adjusted mean of ln (homocysteine) in obesity with LMM group was highest among study groups (all p value < 0.05, Bonferroni post-hoc analysis).

### Association between hyperhomocysteinemia and obesity alone, low muscle mass alone, and coexisting obesity with low muscle mass

Table 3 shows the result of multivariate regression analysis. The impact of the HHcy on having obesity alone, LMM alone, and obesity with LMM was assessed, respectively. In crude model, odd ratios (ORs) (95% confidence interval [CI]) for subjects with HHcys for obesity alone, LMM alone, and obesity with LMM groups compared with control group were 1.057 (1.010–1.106), 1.233 (1.162–1.308), 1.657 (1.323–2.075), respectively. There was a positive trend of ORs from obesity alone, to LMM alone, to obesity with LMM group (*p* for trend < 0.001). In multivariable logistic regression models (model 1 and model) after the adjustment for possible confounding factors, the positive trend remained statistically significant (*p* for trend < 0.001). In model 2, adjusted ORs (95% CI) for subjects with HHcys for obesity alone, LMM alone, and obesity with LMM groups were 0.984 (0.937–1.033), 1.186 (1.117–1.259), and 1.424 (1.134–1.788) compared with control group, respectively (*p* for trend < 0.001), showing the obesity with LMM group the highest ORs for HHcys. Additional information for the results of multivariate logistic analysis in association of each factors with obesity with LMM are expressed in Supplementary Table 1.

**Table 3:** Mutivariate regression analyses showing associations of hyperhomocysteinemia (≥ 15 umol/L) with the presence of obesity and/or low skeletal muscle mass.

	OR (95% CI)		
	Crude	Model 1	Model 2
Control	1 (ref)	1 (ref)	1 (ref)
Obesity alone	1.057 (1.010–1.106)	1.014 (0.969–1.061)	0.984 (0.937–1.033)
LMM alone	1.233 (1.162–1.308)	1.172 (1.105–1.244)	1.186 (1.117–1.259)
Obesity with LMM	1.657 (1.323–2.075)	1.484 (1.183–1.861)	1.424 (1.134–1.788)
*p* for trend	< 0.001	< 0.001	< 0.001

ORs were calculated as the risks of having obesity alone, LMM alone, or obesity with LMM according to the presence of hyperhomocysteinemia.

Model 1: adjusted for age, sex.

Model 2: Model 1 + history of hypertension, history of diabetes, HDL-C, ALT, and CRP.

*ALT* alanine aminotransferase, *CI* confidence interval, *CRP* C-reactive protein, *HDL-C* high-density lipoprotein cholesterol, *LMM* low skeletal muscle mass, *OR* odds ratio.

### Subgroup analysis according to sex and age

The associations of HHcys with obesity alone, LMM alone, obesity with LMM were next examined for subgroups of sex and age in model 3 (Table 4). In both men and women, there was a positive trend in adjusted ORs for the subjects with HHcys in obesity alone, LMM alone, and obesity with LMM groups compared with the control (p for trend < 0.001 in men, 0.012 in women). Interestingly, the associations of HHcys with obesity alone, LMM alone, and obesity with LMM were stronger in women than those in men (*p for* interaction < 0.001). We repeated the analyses for age subgroups (aged < 60 vs ≥ 60). The associations of HHcys with obesity alone, LMM alone, and Obesity with LMM were stronger in older participants (aged ≥ 60) than those in younger participants (< 60 years) (Table 4; *p* for interaction < 0.001).

**Table 4:** Multivariate regression analyses showing associations of hyperhomocysteinemia and the study groups by sex and age.

	Adjusted OR (95% CI)				*p* for trend	*p* for interaction
	Control	Obesity alone	LMM alone	Obesity with LMM		
**Sex**						< 0.001
Men	1 (reference)	0.980 (0.936–1.026)	1.180 (1.112–1.253)	1.464 (1.165–1.840)	< 0.001	
Women	1 (reference)	1.353 (0.902–2.027)	1.338 (0.741–2.417)	4.573 (1.012–20.667)	0.012	
**Age (years)**						< 0.001
< 60	1 (reference)	1.001 (0.951–1.053)	1.174 (1.102–1.251)	1.213 (0.911–1.615)	0.002	
≥ 60	1 (reference)	1.254 (1.028–1.530)	1.875 (1.544–2.277)	2.700 (1.782–4.091)	< 0.001	

Analysis was adjusted for history of hypertension, history of diabetes, HDL-C, ALT, and CRP. Age was adjusted for subgroup analysis according to sex, and the sex was adjusted for the subgroup analysis according to age.

*ALT* alanine aminotransferase, *CI* confidence interval, *CRP* C-reactive protein, *HDL-C* high-density lipoprotein cholesterol, *LMM* low skeletal muscle mass, *OR* odds ratio.

## Discussion

This population-based, cross-sectional study showed that HHcy was independently associated with LMM alone, and obesity with LMM. Furthermore, there was a positive trend for HHcy along the groups from control to obesity alone, LMM alone, and obesity with LMM, which suggests that obesity with LMM is more strongly associated with HHcy than either of condition alone. To the best of our knowledge, this is the first study that demonstrated an association of HHcy with coexistence of obesity with LMM in asymptomatic adults. Through the subgroup analysis, we also found that this association is stronger in women and older participants.

### Association between hyperhomocysteinemia and obesity

While there was a positive trend for HHcy along the groups from control to obesity, LMM, and Obesity with LMM, our study found that there was no independent association between HHcy and obesity. There have been multiple studies evaluating the relationship between Hcy and obesity, but the results were inconsistent. Notably, some of the studies that found positive association between HHcy and obesity were conducted in small number of patients with coexistence of obesity-related disorders^21,22^. In this large-scale study, we initially saw an association between HHcy and obesity in the crude model, but after adjusting for age and sex, this association disappeared and remained insignificant after controlling for further obesity-related disorders such as diabetes and hypertension. This result implicates that obesity per se may not be an independent risk factor, but there may be another underlying mechanism for HHcy seen in obese patients. In fact, Fu et al. demonstrated through meta-analysis that methylentetrahydrofolate reductase gene C677T mutation may drive a positive relationship between HHcy and obesity^23^. Furthermore, other studies suggested that rather than BMI itself, central obesity might be related to HHcy. Wang et al.^14^ found that there was no significant association between HHcy and BMI-based obesity in 11,007 middle-aged Chinese women although circumference-based central obesity was associated with HHcy. Another prospective cohort study performed in 8952 Chinese community residents which included both young and old populations also showed that abdominal obesity was associated with the risk of HHcy for those without cardiovascular comorbidities^24^. We used BMI to define obesity, a measure which cannot capture central obesity, and as a result, we could not further confirm the findings of previous studies. Nonetheless, our study uniquely identified that higher BMI coexisting with LMM is strongly associated with HHcy. Further studies incorporating diverse aspects of obesity and body compositions would be needed to better understand the relationship between HHcy and obesity.

### Association between hyperhomocysteinemia and coexistence of obesity with LMM

Up to date, multiple studies have investigated the association between Hcy and muscle mass or muscle function. In the previous studies, weak hand grip strength^15,16,25^ and LMM^14–16^ were associated with HHcy. However, these studies had small sample sizes with a focus on older adults and did not sufficiently consider potential confounding factors associated with sarcopenia or Hcy levels. A large-scale study recently published from our research group controlled for confounding variables including demographic, inflammatory and metabolic markers, diabetes, and health-behavior factors and found a robust association between HHcy and LMM^12^. Consistent with this finding, our analysis in this study showed an independent association between HHcy and LMM and further found that HHcy may be more strongly associated with Obesity with LMM than LMM or obesity alone.

Only few studies have investigated the relationship between HHcy and LMM with concurrent obesity. Perna et al.^26^ found that there was no significant difference in Hcy level between sarcopenia group and sarcopenic obesity group among 639 hospitalized elderly patients. On the other hand, Park et al.^27^ conducted a prospective cohort study with 2590 Korean adults over 20 years of age and showed that decrease in lean body mass and increase in total body fat proportion were associated with elevation of Hcy level. Although Park et al.^27^ did not examine whether obesity with LMM was associated with greater degree of HHcy than obesity or LMM alone, their analysis provided preliminary evidence that within the same level of lean body mass, higher total body fat proportion was associated with increase in Hcy. Our study builds upon this previous finding by showing that obesity with LMM may be more strongly associated with LMM alone.

Although pathophysiology behind how HHcy can cause LMM or obesity with LMM is not well understood, some animal studies suggest possible mechanisms. In mouse models, HHcy was found to induce diminished proliferative capabilities and increased oxidative stress^28^, decreased large muscle fiber number^29^, and higher susceptibility of skeletal muscle injury and subsequent dysfunction^30^. Likewise, given the independent associations of HHcy with LMM alone and obesity with LMM, HHcy in part could have played a role in the loss of muscle mass. Then, loss of muscle mass and subsequent decrease in physical activity levels may reduce total energy expenditures, which can lead to the accumulation of fat mass, visceral fat in particular^31^. Therefore, Obesity with LMM might be a progression from LMM in which HHcy-induced muscle loss initially occurs, and then accumulation of visceral fat may follow. Given the association between central obesity and HHcy^14,24^, those with concurrent Obesity with LMM might have stronger association with HHcy than LMM alone.

### Subgroup analysis for age and sex

We also performed subgroup analysis to assess whether age-specific or gender-specific differences exist in the associations of HHcy with obesity alone, LMM alone, and obesity with LMM. Our results showed that the association between HHcy and Obesity with LMM was stronger in women and older adults. A possible explanations for this finding among the older adults are impaired Hcy metabolism resulting from the decline of glomerular filtration rate with aging and poor nutrition with low vitamin B levels^13^. Also, it has been reported that loss of muscle mass is accelerated after 60 years of age while fat mass continues to increase until around 75 years of age^3^. Therefore, older adults may have accelerated decline in skeletal muscle and increase in fat mass in the setting of increased Hcy levels.

In terms of gender difference, previous longitudinal studies identified that higher Hcy level was related to lower muscle mass and strength in women^15,16^. Our result is partly consistent with the findings of previous studies in that the relationship between HHcy and Obesity with LMM among women. The reason for this difference is not well understood, but gender-specific hormones might play a role. It has been reported that testosterone is positively while estrogen is inversely correlated with Hcy levels^32^, and reduced estrogen in postmenopausal women have been associated with decline in muscle strength. Moreover, higher prevalence of overweight and obesity defined by BMI and abdominal obesity were noted in older female adults^33^. Thus, older and women adults in particular may be more vulnerable given the age-related increase in Hcy levels and decline of estrogen contributing to further loss of skeletal muscle mass.

### Clinical implication

Increased Hcy may result from various factors such as smoking, alcohol use, caffeine consumption, low vitamin B levels from poor nutrition or gastrointestinal disorders, impaired kidney function or inborn metabolic disorders^13^. Vitamin B12, vitamin B6, and folate are engaged in the intracellular metabolism and elimination of Hcy leading to an inverse relationship between these vitamins and Hcy levels^34–36^. Given this relationship, a few clinical trials investigated the effect of folic acid and vitamin B12 supplementation therapy in delaying muscle strength and physical function decline among older adults^37,38^. While both studies succeeded in lowering Hcy levels in the treatment groups compared to the placebo groups, the supplementation therapy failed to show significant differences in muscle strength and physical function decline between the groups. These results suggest that Hcy level may be a marker of decline in muscle strength rather than a causal factor. Nonetheless, given the previous clinical trials were conducted in elderly who might have other comorbidities that might affect skeletal muscle loss or muscle strength decline, further studies are needed to see whether lowering Hcy would help prevent the development of LMM or Obesity with LMM. Moreover, despite some potential biochemical markers for sarcopenia that have been reported^39^, definite markers of LMM or Obesity with LMM have not yet been found. Therefore, until future clinical studies can confirm causality between HHcy and LMM or Obesity with LMM, levels of Hcy may help clinicians consider for management for LMM and Obesity with LMM.

### Limitation

Our study has several limitations. First, we used BMI to define obesity, but BMI does not reflect the amount of lean mass and fat tissue. This could have affected our result on the relationship between HHcy and obesity. Second, our study was conducted in the Korean population composed of mostly young adults with average age of 38.32 ± 10.8 and 94.6% of men. Since the level of Hcy may be affected by ethnicity^40^, the results of our study may not be generalized to other populations. However, in order to overcome this disproportion of age and sex in the study samples, we performed subgroup analysis which found stronger association of HHcy with Obesity with LMM in older adults and women. Third, because this study was a cross-sectional design, cause-effect associations cannot be inferred. Fourth, the prevalence of hypohomocysteinemia was not assessed in our study population. As far as the authors know, the relationship between hypohomocysteinemia and skeletal muscle mass has been barely studied. Though authors assume that the prevalence of hypohomocysteinemia in our study might be very low due to relatively young and middle-aged participants, this could be a potential factor that can affect the relationship. Lastly, given the observational nature of our study, our results cannot be used to infer causal relationship. Our results could have derived from reverse causality, residual confounding, or both.

## Conclusion

This large-scaled study demonstrated that HHcy was highly associated with coexistence of obesity with LMM, and this association was stronger than either LMM or obesity alone. Furthermore, obesity with LMM in women and older adults had stronger associations with HHcy than those in men and younger adults, respectively. This result suggests HHcy could be a potential biomarker for detecting coexisting obesity with LMM, having a risk for underlying sarcopenic obesity. Therefore, if HHcy is detected in the patient, clinicians may consider a condition of muscle and fat deterioration. Further studies would be needed to investigate pathophysiologic pathways of HHcys on muscle and fat composition.

## Materials and methods

### Study subjects and study period

We conducted a population-based cross-sectional study using a subsample of the Kangbuk Samsung Health Study (KSHS). KSHS is a cohort study of Korean population who had an annual or biennial health checkup program^41,42^. Data was collected at the first-visit health examination data of the participant. The results of examinations, laboratory analysis, answers to the standardized questionnaire from each health check-up of the study participants were stored in the KSHS database. The data used in our study was extracted from the database.

**Figure 1:**
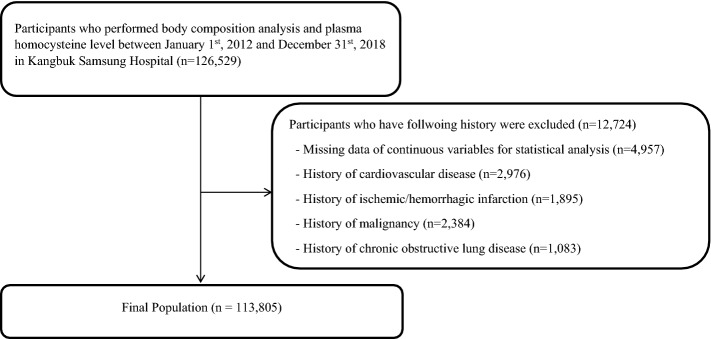
Flow diagram of the study population.

To briefly summarize how the database was formed, the study participants were recruited from one of the Kangbuk Samsung Hospital Total Healthcare Centers in Seoul and Suwon, Republic of Korea from January 1, 2012 and December 31, 2018 (*n* = 126,529). All the participants were examined for body composition analysis and total plasma Hcy value. We excluded participants with following histories: cardiovascular disease, stroke, chronic obstructive pulmonary disease or chronic lung disease, cancer, due to their known independent association between plasma Hcy level^43–46^. Participants with missing values for confounding variables (age, sex, history of hypertension, history of diabetes, high-density lipoprotein cholesterol (HDL-C), alanine aminotransferase (ALT) and C-reactive protein (CRP) were also excluded from the analysis to increase the accuracy of the analysis. Among the participants, participants with history of cardiovascular disease (*n* = 2976), history of ischemic/hemorrhagic infarction (*n* = 1895), history of malignancy (*n* = 2384), history of chronic obstructive lung disease (*n* = 1083), and participants with missing data of continuous variables for statistical analysis (*n* = 4957) were excluded. After the exclusion (*n* = 12,724), total 113,805 participants were included for the final analysis (Fig. 1).

Our study protocol was approved by the institutional review board (IRB) of Kangbuk Samsung Hospital (IRB no. KBSMC 2021-11-036). This study was conducted in accordance with the 1975 Declaration of Helsinki. The requirement for the informed consent was waived by the IRB of Kangbuk Samsung Hospital since we used unidentifiable datasets that were collected as part of the routine health checkup.

### Measurements

Data on medical history of hypertension, diabetes, ischemic/hemorrhagic infarction, cardiovascular disease, and malignancy were collected by the examining physicians using standardized, self-administered questionnaires^47^. Anthropometric measurements including blood pressure (mmHg), height (cm), weight (kg), waist circumference (cm), body mass index (BMI) (kg/m^2^) were done by a trained medical staff or nurse. Blood sample was collected at antecubital vein after at least 8 h of fasting to perform the laboratory analysis for estimating serum Hcy level (mml/L), HDL-C (mg/dL), ALT (IU/L), and CRP (mg/dL).

Serum homocysteine was measured by an enzymatic assay using automated chemistry analyzer Modular DPP (Roche Diagnostics, Tokyo, Japan) from 2012 to 2014 and cobas 8000 c702 from 2015 to 2018. The intra-assay coefficients of variation for quality control specimens of lower levels and higher levels were 1.56–3.77% and 1.02–2.47% during study period. Following previous literature^48^, serum Hcy concentration greater than 15 umol/L was defined as HHcy.

Appendicular skeletal muscle mass (kg), the sum of muscle mass of extremities was measured using bioelectrical impedance analysis (BIA, InBody 720, Biospace, South Korea). Previous study reported BIA predicts valid estimates of skeletal muscle mass in adults^49^. We calibrated BIA everyday morning before we initiated the test and validated accuracy and reproducibility of the device. Skeletal muscle mass index (SMI) was measured by dividing the appendicular skeletal muscle mass (kg) by the square of the height (m^2^)^50,51^.

Laboratory medicine department of Kangbuk Samsung Hospital in Seoul, Korea has been validated by the Korean Society of Laboratory Medicine and Korean Association of Quality Assurance for Clinical laboratories. Additionally, the laboratory was accredited by the College of American Pathologist’s Proficiency Testing program.

### Classifications of participants

Subjects were classified into four groups according to the presence of obesity and/or LMM. Obesity was defined as body mass index ≥ 25 (kg/m^2^) according to the World Health Organization recommendation for the Asian-Pacific region^52^. LMM was determined by SMI (skeletal muscle mass index) value less than 2 SD below the sex-specific mean of healthy young adults (age 18–39 years)^51^. Gender specific cut-off values for LMM in men were 7.39 kg/m^2^, in women was 5.43 kg/m^2^, respectively. The subjects were classified into ‘control’, ‘obesity alone’, ‘LMM alone’, and ‘obesity with LMM’ groups according to these definitions.

### Statistical analysis

We used one-way analysis of variance (ANOVA) to compare continuous variables and chi-square test for categorical variables. Prevalence (%) of HHcy in control, obesity, LMM, obesity with LMM group was compared by Chi-square test and post-hoc analysis, using Bonferroni method for correction. Multivariable logistic regression analyses were used to analyze the association between HHcy and each group. In the analyses, the independent variable was the HHcy expressed as a binominal categorical variable according to the presence or absence of HHcy. Dependent variables were also expressed as binominal categorical variables according to the presence or absence of obesity and/or LMM. We analyzed the association using three different models to adjust confounding factors: Crude model; Model 1 adjusted for age, sex; Model 2 adjusted for age, sex, history of hypertension, history of diabetes, HDL-C, ALT and CRP. We selected confounding factors based on previous literature that proved their association with LMM alone, obesity alone, and coexisting obesity with LMM^53–63^.

We used ORs to calculate the risk of having HHcy in each group, and 95% CI was calculated. For model 2, we additionally performed subgroup analysis by stratifying the participants based on the sex and age (≥ 60 and < 60 year) to elucidate the association in different sex and age groups, considering sex- and age- specific differences in body composition and prevalence of sarcopenic obesity^64^. In addition, we conducted an analysis to assess the relationships when Hcy was expressed as a continuous variable. Due to positively skewed distribution of plasma Hcy level, Hcy values were natural log-transformed (ln) for best fitting model and analyzed as a continuous variable. Finally, adjusted means of Ln (Hcy) value in each group was assessed by using analysis of covariance (ANCOVA) in model 3. For statistical analysis, a two-tailed *p*-value < 0.05 were considered significant. We used IBM SPSS version 26.0 (IBM Co., NU, USA) for all statistical analysis.

## Supplementary Information

Supplementary Information.

## Data Availability

The datasets of the current study cannot be made openly available to protect the medical information of participants. However, the corresponding author can provide the dataset on a reasonable request.

## References

[1] Cruz-Jentoft AJ (2010). Sarcopenia: European consensus on definition and diagnosis: Report of the European Working Group on Sarcopenia in Older People. Age Ageing.

[2] Janssen I, Ross R (2005). Linking age-related changes in skeletal muscle mass and composition with metabolism and disease. J. Nutr. Health Aging.

[3] Kyle UG (2001). Age-related differences in fat-free mass, skeletal muscle, body cell mass and fat mass between 18 and 94 years. Eur. J. Clin. Nutr..

[4] Landi F (2012). Sarcopenia as a risk factor for falls in elderly individuals: Results from the ilSIRENTE study. Clin. Nutr..

[5] Edwards MH, Dennison EM, Aihie Sayer A, Fielding R, Cooper C (2015). Osteoporosis and sarcopenia in older age. Bone.

[6] Petermann-Rocha F (2020). New versus old guidelines for sarcopenia classification: What is the impact on prevalence and health outcomes?. Age Ageing.

[7] Tsekoura M, Kastrinis A, Katsoulaki M, Billis E, Gliatis J (2017). Sarcopenia and its impact on quality of life. Adv. Exp. Med. Biol..

[8] Atkins JL, Wannamathee SG (2020). Sarcopenic obesity in ageing: Cardiovascular outcomes and mortality. Br. J. Nutr..

[9] Kob R (2015). Sarcopenic obesity: Molecular clues to a better understanding of its pathogenesis?. Biogerontology.

[10] Yoo JH (2020). Effects of low skeletal muscle mass and sarcopenic obesity on albuminuria: A 7-year longitudinal study. Sci. Rep..

[11] Kim SJ, Lim KS, Song MS, Kang Y, Lee SY (2009). Prevalence of hyperhomocysteinemia and related factors in a community-based health examination survey: A cross-sectional study. J. Prev. Med. Public Health.

[12] Choi JH (2022). Association between elevated plasma homocysteine and low skeletal muscle mass in asymptomatic adults. Endocrinol. Metab. (Seoul).

[13] De Giuseppe R (2021). Sarcopenia and homocysteine: Is there a possible association in the elderly? A narrative review. Nutr. Res. Rev..

[14] Wang Y (2019). Central but not general obesity is positively associated with the risk of hyperhomocysteinemia in middle-aged women. Nutrients.

[15] Vidoni ML, Pettee Gabriel K, Luo ST, Simonsick EM, Day RS (2018). Relationship between homocysteine and muscle strength decline: The Baltimore longitudinal study of aging. J. Gerontol. A Biol. Sci. Med. Sci..

[16] Lee WJ, Peng LN, Loh CH, Chen LK (2020). Sex-different associations between serum homocysteine, high-sensitivity C-reactive protein and sarcopenia: Results from I-Lan longitudinal aging study. Exp. Gerontol..

[17] Elshorbagy AK (2008). Homocysteine, cysteine, and body composition in the hordaland homocysteine study: Does cysteine link amino acid and lipid metabolism?. Am. J. Clin. Nutr..

[18] Atkins JL, Whincup PH, Morris RW, Wannamethee SG (2014). Low muscle mass in older men: The role of lifestyle, diet and cardiovascular risk factors. J. Nutr. Health Aging.

[19] Walston JD (2012). Sarcopenia in older adults. Curr. Opin. Rheumatol..

[20] Stevens J (1998). The effect of age on the association between body-mass index and mortality. N. Engl. J. Med..

[21] Nervana MK, Bayoumy A, El-Shabrawi MM, Atwa KA (2012). Assessment of homocysteine plasma levels and insulin resistance among obese women with anovulatory infertility. Life Sci. J..

[22] Karatela RA, Sainani GS (2009). Plasma homocysteine in obese, overweight and normal weight hypertensives and normotensives. Indian Heart J..

[23] Fu L, Li YN, Luo D, Deng S, Hu YQ (2019). Plausible relationship between homocysteine and obesity risk via MTHFR gene: A meta-analysis of 38,317 individuals implementing Mendelian randomization. Diabetes Metab. Syndr. Obes..

[24] Xiang Y (2021). Association of obesity with the risk of hyperhomocysteinemia among the Chinese community residents: A prospective cohort study in Shanghai, China. Nutrients.

[25] Swart KM (2013). Homocysteine and the methylenetetrahydrofolate reductase 677C–>T polymorphism in relation to muscle mass and strength, physical performance and postural sway. Eur. J. Clin. Nutr..

[26] Perna S (2017). Sarcopenia and sarcopenic obesity in comparison: Prevalence, metabolic profile, and key differences. A cross-sectional study in Italian hospitalized elderly. Aging Clin. Exp. Res..

[27] Park SB, Georgiades A (2013). Changes in body composition predict homocysteine changes and hyperhomocysteinemia in Korea. J. Korean Med. Sci..

[28] Veeranki S, Lominadze D, Tyagi SC (2015). Hyperhomocysteinemia inhibits satellite cell regenerative capacity through p38 alpha/beta MAPK signaling. Am. J. Physiol. Heart Circ. Physiol..

[29] Veeranki S, Winchester LJ, Tyagi SC (2015). Hyperhomocysteinemia associated skeletal muscle weakness involves mitochondrial dysfunction and epigenetic modifications. Biochim. Biophys. Acta.

[30] Singh M (2021). High-methionine diet in skeletal muscle remodeling: Epigenetic mechanism of homocysteine-mediated growth retardation. Can. J. Physiol. Pharmacol..

[31] Clifton PM, Keogh JB, Noakes M (2008). Long-term effects of a high-protein weight-loss diet. Am. J. Clin. Nutr..

[32] Dierkes J (2001). Factors explaining the difference of total homocysteine between men and women in the European Investigation Into Cancer and Nutrition Potsdam study. Metabolism.

[33] Sardinha LB (2012). Prevalence of overweight, obesity, and abdominal obesity in a representative sample of Portuguese adults. PLoS ONE.

[34] Ganji V, Kafai MR (2006). Population reference values for plasma total homocysteine concentrations in US adults after the fortification of cereals with folic acid. Am. J. Clin. Nutr..

[35] Selhub J (1999). Serum total homocysteine concentrations in the third National Health and Nutrition Examination Survey (1991–1994): Population reference ranges and contribution of vitamin status to high serum concentrations. Ann. Intern. Med..

[36] Clarke R (2004). Vitamin B12 and folate deficiency in later life. Age Ageing.

[37] Lewerin C (2005). Significant correlations of plasma homocysteine and serum methylmalonic acid with movement and cognitive performance in elderly subjects but no improvement from short-term vitamin therapy: A placebo-controlled randomized study. Am. J. Clin. Nutr..

[38] Swart KM (2016). A randomized controlled trial to examine the effect of 2-year vitamin B12 and folic acid supplementation on physical performance, strength, and falling: Additional findings from the B-PROOF study. Calcif. Tissue Int..

[39] Cesari M (2012). Biomarkers of sarcopenia in clinical trials-recommendations from the International Working Group on Sarcopenia. J. Cachexia Sarcopenia Muscle.

[40] Guo S (2015). Ethnic differences in the prevalence of high homocysteine levels among low-income rural Kazakh and Uyghur adults in far western China and its implications for preventive public health. Int. J. Environ. Res. Public Health.

[41] Chang Y (2019). Alcoholic and non-alcoholic fatty liver disease and associations with coronary artery calcification: Evidence from the Kangbuk Samsung Health Study. Gut.

[42] Lee M-K (2015). Higher association of coronary artery calcification with non-alcoholic fatty liver disease than with abdominal obesity in middle-aged Korean men: The Kangbuk Samsung Health Study. Cardiovasc. Diabetol..

[43] Kalra DK (2004). Homocysteine and cardiovascular disease. Curr. Atheroscler. Rep..

[44] Zhao M (2017). Homocysteine and stroke risk: Modifying effect of methylenetetrahydrofolate reductase C677T polymorphism and folic acid intervention. Stroke.

[45] Seemungal TA (2007). Plasma homocysteine is elevated in COPD patients and is related to COPD severity. Int. J. Chron. Obstruct. Pulmon. Dis..

[46] Hasan T (2019). Disturbed homocysteine metabolism is associated with cancer. Exp. Mol. Med..

[47] Devaux M, Sassi F (2015). Alcohol consumption and harmful drinking: Trends and social disparities across OECD countries. OECD Health Work. Pap..

[48] Zhang Z (2016). Combined effect of hyperhomocysteinemia and hypertension on the presence of early carotid artery atherosclerosis. J. Stroke Cerebrovasc. Dis..

[49] Janssen I, Heymsfield SB, Baumgartner RN, Ross R (2000). Estimation of skeletal muscle mass by bioelectrical impedance analysis. J. Appl. Physiol..

[50] Fukuoka Y (2019). Importance of physical evaluation using skeletal muscle mass index and body fat percentage to prevent sarcopenia in elderly Japanese diabetes patients. J. Diabetes Investig..

[51] Janssen I, Heymsfield SB, Ross R (2002). Low relative skeletal muscle mass (sarcopenia) in older persons is associated with functional impairment and physical disability. J. Am. Geriatr. Soc..

[52] Oh SW (2011). Obesity and metabolic syndrome in Korea. Diabetes Metab. J..

[53] Bai T (2020). Sarcopenia is associated with hypertension in older adults: A systematic review and meta-analysis. BMC Geriatr..

[54] Khadra D (2019). Association between sarcopenic obesity and higher risk of type 2 diabetes in adults: A systematic review and meta-analysis. World J. Diabetes.

[55] Lu CW (2013). Sarcopenic obesity is closely associated with metabolic syndrome. Obes. Res. Clin. Pract..

[56] Kreidieh D (2018). Association between sarcopenic obesity, type 2 diabetes, and hypertension in overweight and obese treatment-seeking adult women. J. Cardiovasc. Dev. Dis..

[57] Bekkelund SI, Jorde R (2019). Alanine aminotransferase and body composition in obese men and women. Dis. Mark..

[58] Schrager MA (2007). Sarcopenic obesity and inflammation in the InCHIANTI study. J. Appl. Physiol..

[59] Choi KM (2016). Sarcopenia and sarcopenic obesity. Korean J. Intern. Med..

[60] Du Y (2019). Sex differences in the prevalence and adverse outcomes of sarcopenia and sarcopenic obesity in community dwelling elderly in East China using the AWGS criteria. BMC Endocr. Disord..

[61] Moon SS (2014). Low skeletal muscle mass is associated with insulin resistance, diabetes, and metabolic syndrome in the Korean population: The Korea National Health and Nutrition Examination Survey (KNHANES) 2009–2010. Endocr. J..

[62] Han JM, Lee MY, Lee KB, Kim H, Hyun YY (2020). Low relative skeletal muscle mass predicts incident hypertension in Korean men: A prospective cohort study. J. Hypertens..

[63] Mesinovic J, Zengin A, De Courten B, Ebeling PR, Scott D (2019). Sarcopenia and type 2 diabetes mellitus: A bidirectional relationship. Diabetes Metab. Syndr. Obes..

[64] Wagenaar CA, Dekker LH, Navis GJ (2021). Prevalence of sarcopenic obesity and sarcopenic overweight in the general population: The lifelines cohort study. Clin. Nutr..

