# Supplementary material for: Relationship between hyperhomocysteinemia and coexisting obesity with low skeletal muscle mass in asymptomatic adult population
Source: Sci Rep. 2022 Jul 20;12:12439. doi: 10.1038/s41598-022-16401-1 (PMC9300668; doi:10.1038/s41598-022-16401-1)
Supplement: Supplementary file 1 — Supplementary Information. [file 41598_2022_16401_MOESM1_ESM.docx]

**Supplementary Data.**

**

**

**Supplementary Fig. 1** Comparison of Natural log transformed homocysteine level in control, obesity alone, LMM alone, and Obesity with LMM group. Adjusted means of ln (homocysteine) level in the groups were estimated from ANCOVA after adjustments for age, sex, history of hypertension, history of diabetes, HDL-C, ALT, and CRP.

^*^Adjusted p<0.05 versus control group in post hoc analysis.

^†^Adjusted p<0.05 versus obesity alone group in post hoc analysis.

^‡^Adjusted p<0.05 versus LMM alone group in post hoc analysis.

^#^Adjusted p<0.05 versus obesity with LMM group in post hoc analysis.

ALT alanine aminotransferase, CRP C-reactive protein, HDL-C high-density lipoprotein cholesterol, LMM low skeletal muscle mass.

**Supplementary Table 1**. Association of factors with coexistence of obesity and low skeletal muscle mass using multivariate regression analysis

| Variables | Adjusted Odds Ratio | 95% CI | *p* value |
| --- | --- | --- | --- |
| **HHcy** | **1.424** | **1.134-1.788** | **0.002** |
| Age | 1.051 | 1.045-1.058 | <0.001 |
| Male | 5.037 | 3.178-7.984 | <0.001 |
| HTN | 1.901 | 1.564-2.312 | <0.001 |
| DM | 1.221 | 0.914-1.630 | 0.176 |
| ALT | 1.031 | 1.028-1.033 | <0.001 |
| CRP | 1.542 | 1.389-1.712 | <0.001 |
| HDL-C | 0.979 | 0.973-0.985 | <0.001 |

Analysis was adjusted for age, sex, history of hypertension, history of diabetes, HDL-C, ALT, and CRP. Age was adjusted for subgroup analysis according to sex, and the sex was adjusted for the subgroup analysis according to age.

CI, confidence interval; HHcy, hyperhomocysteinemia; HTN, hypertension; DM, diabetes mellitus; ALT, alanine aminotransferase; CRP, C-reactive protein; HDL-C, high-density lipoprotein cholesterol
